# Randomized Trial of High Dose Oral Rifampicin in Adult Tuberculous Meningitis

**DOI:** 10.1056/NEJMoa2502866

**Published:** 2025-12-18

**Authors:** David B Meya, Fiona V Cresswell, Biyue Dai, Nicole Engen, Kogieleum Naidoo, Ahmad Rizal Ganiem, Darma Imran, Mable Kabahubya, Richard J Lessells, Vycke Yunivita, Riwanti Estiasari, Lillian Tugume, Bongeka Hlabisa, Media Yuni Kurniawati, Noveline Sagita, Enock Kagimu, Kartika Maharani, Jane Gakuru, Maula N. Gaharu, Timothy Mugabi, Sarah Kimuda, Suzan Namombwe, Lindsey te Brake, Rob Aarnoutse, Elin M Svensson, Ananta S Bangdiwala, Sylvia Namanda, Nathan C Bahr, Abdu K Musubire, Mahomed Yunus Suleman Moosa, Raph L. Hamers, Suzaan Marais, David R Boulware, Reinout van Crevel, Rovina Ruslami

**Affiliations:** 1 Infectious Diseases Institute, College of Health Sciences, Makerere University, P.O Box 22418, Kampala, Uganda; 2 Division of Infectious Diseases & International Medicine, Department of Medicine, University of Minnesota, Minneapolis, MN, 55455, USA; 3 Centre for Global Health Research, Brighton and Sussex Medical School, Brighton, UK; 4 Clinical Research Department, London School of Hygiene & Tropical Medicine, London, WC1E 7HT, UK; 5 Division of Biostatistics & Health Data Science, School of Public Health, University of Minnesota, Minneapolis, MN, 55455, USA; 6 Centre for the AIDS Programme of Research in South Africa (CAPRISA), Doris Duke Medical Research Institute, Durban, 4041, South Africa; 7 SAMRC-CAPRISA HIV-TB Pathogenesis and Treatment Research Unit, Doris Duke Medical Research Institute, University of KwaZulu Natal, Durban, South Africa; 8 Department of Neurology, Faculty of Medicine, Universitas Padjadjaran / Hasan Sadikin Hospital, Bandung, 40161, Indonesia; 9 Research Center for Care and Control of Infectious Disease Universitas Padjadjaran, Bandung, 40161, Indonesia; 10 Department of Neurology, Faculty of Medicine Universitas Indonesia, Cipto Mangunkusumo Hospital, Jakarta 10430, Indonesia; 11 KwaZulu-Natal Research Innovation and Sequencing Platform, School of Laboratory Medicine & Medical Sciences, University of KwaZulu-Natal, Durban, 4001, South Africa; 12 Department of Biomedical Sciences, Faculty of Medicine, Universitas Padjadjaran, Bandung, 40161, Indonesia; 13 Department of Neurology, Cibabat General Hospital, Cimahi, 40513, Indonesia; 14 Faculty of Medicine, Bandung Islamic University, Bandung, 40116, Indonesia; 15 Department of Neurology, Immanuel Hospital, Bandung, 40233, Indonesia; 16 Department of Neurology, Bhayangkara TK. I Pusdokkes Polri Hospital, Jakarta, Indonesia; 17 Department of Pharmacy, Pharmacology and Toxicology, Radboud Institute for Medical Innovation, Radboud University Medical Centre, Nijmegen, The Netherlands; 18 Department of Pharmacy, Uppsala University, Uppsala, Sweden; 19 Department of Infectious Diseases, Division of Internal Medicine, Nelson R Mandela School of Medicine, University of KwaZulu-Natal, Durban, 4013, South Africa; 20 Oxford University Clinical Research Unit Indonesia, Faculty of Medicine Universitas Indonesia, Jakarta, 10430, Indonesia; 21 Centre for Tropical Medicine and Global Health, Nuffield Department of Medicine, University of Oxford, Oxford, UK; 22 Division of Neurology, Department of Medicine, University of Cape Town & Neurology Research Group, Neuroscience Institute, University of Cape Town, Cape Town; 23 Department of Internal Medicine, Radboud University Medical Centre, Nijmegen, The Netherlands; 24 Blizard Institute, Faculty of Medicine & Dentistry, Queen Mary University of London, London E1 2AT, UK

**Keywords:** Tuberculosis, Meningeal, Tuberculous meningitis, rifampin, Randomized Controlled Trial

## Abstract

**Background:**

Tuberculous meningitis is often lethal, and many survivors suffer from disabilities despite antimicrobial treatment and adjunctive corticosteroids. Standard-dose rifampicin has limited central nervous system penetration. We hypothesized that intensified higher-dose rifampicin could improve survival.

**Methods:**

We performed a randomized, double-blind, placebo-controlled clinical trial in adults with tuberculous meningitis in Indonesia, South Africa and Uganda. We enrolled persons with or without HIV co-infection to standard daily isoniazid, rifampicin, ethambutol, and pyrazinamide plus additional rifampicin (total 35 mg/kg) or matched-placebo (total 10 mg/kg) for the first 8 weeks. The primary endpoint was 6-month survival.

**Results:**

We randomized 499 participants for intent-to-treat analysis, of whom 304 (61%) were people living with HIV, and 428 (86%) had confirmed or probable tuberculous meningitis. During six months follow-up, 109 of 249 participants (Kaplan-Meier estimate: 44.6%) died in the high-dose rifampicin group and 100 of 250 (Kaplan-Meier estimate: 40.7%) died in the standard-dose group (hazard ratio, 1.17; 95% confidence interval, 0.89-1.54; p=0.25). Among participants who died by 6-months, the median time to death was 13 (interquartile range, 4 to 39) days with high-dose versus 24.5 (interquartile range, 6 to 56) days with standard-dose. Excess mortality occurred with high-dose group among those with baseline cerebrospinal fluid <5 white cells/mm^3^ or receiving HIV therapy. We did not observe differences in safety. Drug-induced liver injury occurred in 8.0% with high-dose and 4.4% with standard-dose rifampicin but without deaths occurring.

**Conclusions:**

There is no evidence of beneficial effect from high-dose rifampicin for tuberculous meningitis, and the potential for a harmful effect cannot be ruled out.

(Supported by MRC, and others; Clinical Trial numberISRCTN15668391)

Tuberculous meningitis represents a severe manifestation of tuberculosis.^1,2^ Despite guidelines recommending isoniazid, pyrazinamide, ethambutol, and rifampicin (10 mg per kilogram/day), outcomes for tuberculous meningitis remain poor, with mortality as high as 50% among persons with HIV.^3^ Rifampicin, a cornerstone drug for tuberculous meningitis, as evidenced by the higher mortality in rifampicin-resistant disease,^4^ has limited penetration into cerebrospinal fluid (CSF) achieving ~5% of plasma levels.^5^ Consequently, standard rifampicin dosing achieves undetectable CSF concentrations for the majority of persons,^6–8^ raising concerns over the adequacy of standard rifampicin dosing in tuberculous meningitis.

Emerging evidence suggests that higher rifampicin doses may improve tuberculous meningitis treatment outcomes.^9^ Four phase II trials have shown a dose-exposure relationship for rifampicin, with higher doses enhancing plasma and CSF drug concentrations.^6–8,10^ A Vietnamese trial of 817 persons with tuberculous meningitis testing 15 mg per kilogram oral rifampicin showed no benefit.^2^ However, a meta-analysis of phase II Indonesian trials indicated that rifampicin doses of 30 mg per kilogram daily, resulting in 4.7-fold higher total (protein-unbound plus bound) rifampicin plasma exposure, were associated with a 40% higher 6-month survival, albeit the uncertainty in the estimate of the effect was large (relative standard error 86%).^5^ Given mixed evidence, a well powered randomized clinical trial evaluating high-dose rifampicin was deemed necessary.

We report the outcomes of a multi-country trial evaluating the effectiveness of high-dose oral rifampicin on survival in adults with tuberculous meningitis. We hypothesized that a daily dose of ~35 mg per kg of oral rifampicin given for 8 weeks, combined with the standard anti-tuberculosis regimen, would improve 6-month survival compared with the standard ~10 mg per kg rifampicin dose.

## Methods

### Study Design

The “High Dose Oral Rifampicin to Improve Outcomes from Adult Tuberculous Meningitis” (HARVEST) trial was a double-blind, placebo-controlled, randomized phase III trial with parallel group design, conducted in Indonesia, South Africa, and Uganda (ISRCTN15668391).^11^ Research ethics committees and relevant regulatory approvals occurred at all sites.

### Study population and setting

The trial aimed to enroll 500 eligible participants aged ≥18 years with tuberculous meningitis at 9 hospitals. Eligibility criteria included microbiologically-confirmed (positive CSF Xpert MTB/Rif Ultra (Cepheid, CA),^12,13,14^ mycobacterial growth indicator tube culture (Becton Dickinson, NJ), or smear microscopy) or suspected tuberculous meningitis based on abnormal CSF parameters and clinical findings with antituberculous treatment planned. Written informed consent was obtained from all participants, or surrogates for people lacking capacity to consent. Exclusion criteria included hypersensitivity, receipt of >5 doses of antituberculous therapy, confirmed untreated neuroinfection other than tuberculosis, HIV protease inhibitor use, corticosteroid contraindication, pregnancy or breastfeeding, or inability to follow-up. As meningitis is a medical emergency and similar to other meningitis trials,^16^ participants could be randomized immediately and withdrawn within 72 hours for baseline late exclusion criteria (alanine transaminase >5 times the upper limit of normal or glomerular filtration rate <30 ml/min/1.73m^2^). Participants could be withdrawn within 15 days for rifampicin resistance or confirmed alternative neuroinfection with decision to stop antituberculous therapy. Full details of study conduct can be found in the protocol at nejm.org.

Participants were classified by the uniform case definition as definite, probable, or possible tuberculous meningitis.^15^

### Randomization and Blinding

We randomized participants in a 1:1 ratio to either high-dose or standard-dose oral rifampicin, using permutated blocks with sizes of two or four. Randomization was stratified by trial centers, HIV status, and tuberculous meningitis severity (Medical Research Council grade one versus grades two or three). Both participants and investigators were blinded to treatment allocation, with identical-appearing intervention and placebo tablets.

### Intervention

Participants in the intervention arm received high-dose oral rifampicin of ~35 mg per kilogram daily, administered for 8 weeks. This dose was achieved by providing extra rifampicin tablets (900 mg if ≤38 kilograms, otherwise 1200 mg) in addition to the standard tuberculosis fixed-dose combination tablets that included rifampicin (~10 mg per kilogram), isoniazid, pyrazinamide, and ethambutol.^17^ Participants in the control arm received the same standard tuberculosis fixed-dose combination tablets that included rifampicin (~10 mg per kilogram) with the same number of placebo tablets as the intervention group. Participants received adjunctive corticosteroids as per international guidelines,^17^ with clinician discretion (Supplementary Methods).

### Outcomes

The primary outcome was 6-month survival. Secondary outcomes included 12-month survival, improvement in functional status as measured by the modified Rankin scale at week 24; Liverpool outcome score at 2-weeks and 6-months; neurocognitive performance at 2 and 12 months;^18,19^ hospitalization duration; incidence of all-cause treatment discontinuation for ≥5 days in the first 8-weeks; re-hospitalization due to neurological decline; and incidence of grade ≥3 or serious adverse events, including hepatotoxicity.

### Follow-up and Data Collection

Following randomization, participants were monitored daily until hospital discharge. Following hospital discharge, clinical and laboratory assessments occurred at 2, 4, and 8-weeks, with additional follow-up visits at weeks-12, 18, 24, 36, and 52. An independent data safety monitoring board reviewed interim data at least annually.

### Statistical Analysis

Assuming 50% survival in the control group, an overall sample size of 500 participants (250 per group) provided 80% power with a two-sided 0.05 significance level to detect a hazard ratio for death of 0.68 (approximately 13% absolute survival improvement) while accounting for ≤5% loss-to-follow up.

The primary analysis was based on the intention-to-treat population, which was pre-specified to include all randomized participants who were alive at the time of randomization, excluding study-sponsored withdrawals including late exclusion criteria. Survival distributions and 6-month mortality were estimated via Kaplan-Meier estimators. The treatment effect of high-dose rifampicin vs standard-dose rifampicin was estimated via hazard ratio from an unadjusted Cox regression. Adjusted analyses were conducted with pre-specified covariates, including randomization strata, age, gender, baseline Glasgow coma scale (<15 or 15) and tuberculous meningitis case definition. Additional statistical details are included in Supplementary Methods. Predefined subgroup analyses examined treatment effect across key clinical and demographic subgroups: baseline Glasgow coma scale score, severity grade, HIV status, antiretroviral status, and clinical trial site. Subgroup analyses were not adjusted for multiplicity.

For secondary endpoints, the modified Rankin Scale at week-24 was compared between the treatment arms using a cumulative logistic regression with proportional odds assumption. For safety endpoints, the total number of events as well as the total number and percentage of participants reporting at least one event were summarized by arms. Fine-Gray model accounted for death as a competing risk for rehospitalization.

## Results

### Participants

**Figure 1:**
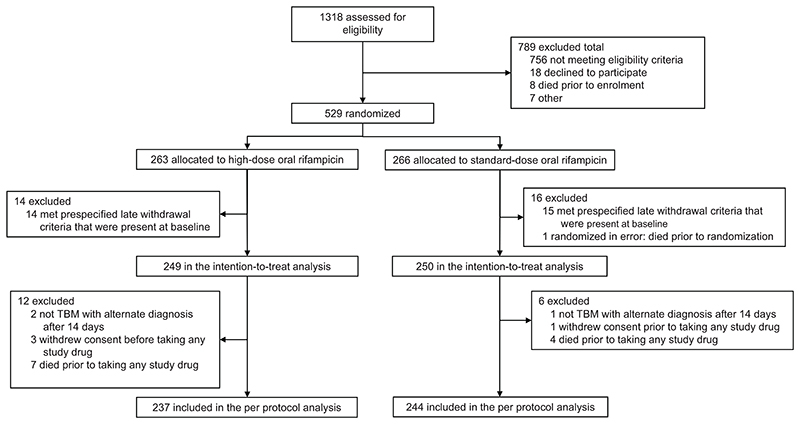
CONSORT Diagram Overall, 529 participants were randomized of whom 29 met late exclusion criteria present at baseline and were administratively withdrawn. Table S1 in the Supplementary Appendix lists screening and enrollment by site. Table S2 in the Supplementary Appendix lists exclusion and late withdrawal criteria. One participant was discovered to have died in the period between informed consent and randomization, resulting in 499 participants included in the intent-to-treat analysis. Eight persons in each arm withdrew consent for the intervention.

From March 12, 2021, to July 31, 2024, we randomly assigned 529 adult patients with tuberculous meningitis to receive either high-dose or standard-dose oral rifampicin. Of these participants, 30 (5.6%) were excluded by baseline late exclusion criteria, leaving 499 participants (249 in the high-dose group and 250 in the standard-dose group) in the intention-to-treat population (Figure 1). Study visit attendance through month 6 of follow-up was 96%, and adherence to study treatment was good, with 6% missing ≥2 days of study drug or matched-placebo through 8 weeks.

### Baseline characteristics

Participant characteristics were similar between the trial groups (Table 1). The median age was 37 years (interquartile range, 28 to 45), and 222 of 499 (44%) were female. Overall, 306 (61%) participants had Medical Research Council grade 2 severity, and 218 (43.7%) had microbiologically-confirmed definite tuberculous meningitis. Overall, 304 of 499 (61%) were persons living with HIV; 125 of 304 (41%) were receiving antiretroviral therapy, and 115 of 226 (51%) had CD4 counts <100 cells/mm^3^. At randomization, 348 of 499 (70%) had received antituberculous treatment for a median of 3 (interquartile range, 1 to 4) days. Overall, 473 of 499 (95%) were prescribed corticosteroids.

### Primary Outcome

**Figure 2:**
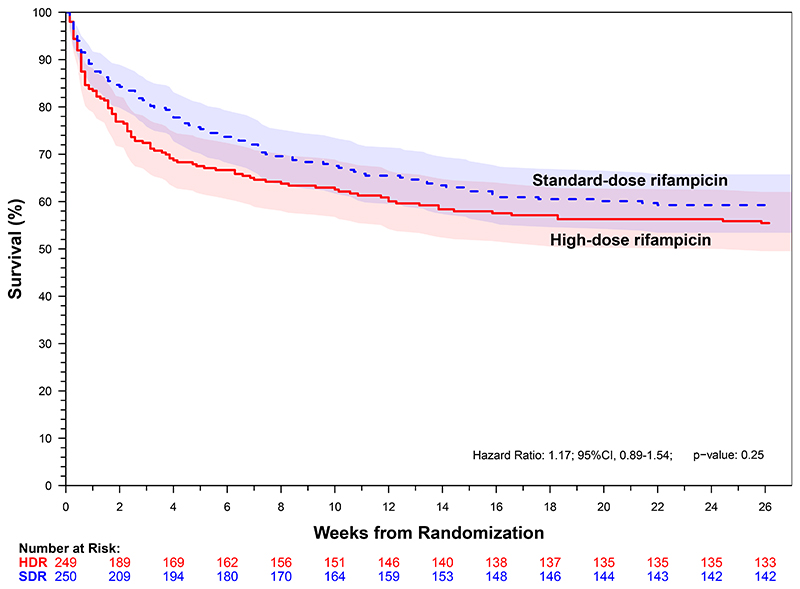
Kaplan–Meier Curves for 6-month Survival by Treatment Group and Key Subgroups The overall 6-month mortality estimated via Kaplan-Meier approach was 44.6% (95% CI: 37.9% to 50.5%) for the high-dose 35 mg per kilogram rifampicin group and 40.7% (95% CI 34.2% to 46.6%) for the standard-dose 10 mg per kilogram rifampicin group as displayed in Panel A (Hazard Ratio, 1.17; 95% confidence interval, 0.89 to 1.54; P = 0.25). Panel B displays the between-group differences in survival over time. In the first 21 days, the high-dose group had 68 deaths (Kaplan-Meier estimate: 27.6%, 95% CI: 21.8% to 33.0%) compared with 48 deaths (Kaplan-Meier estimate: 19.4%, 95% CI: 14.3% to 24.2%) in the standard-dose group. Eight participants in each group were censored before 6 months (range 1 to 162 days). Panels C display the outcomes by HIV status.

Death occurred in 109 of 249 (Kaplan-Meier estimate: 44.6%) participants within six months in the high-dose rifampicin group and in 100 of 250 (Kaplan-Meier estimate: 40.7%) in the standard-dose group (Hazard Ratio, 1.17; 95% confidence interval [CI], 0.89 to 1.54; P=0.25; Figure 2). Similar results were observed in the adjusted analysis with pre-specified baseline covariates or using per-protocol population. Among participants who died within 6 months, the median time to death in the high-dose group was 13 (interquartile range, 4 to 39) days and 24.5 (interquartile range, 6 to 56) days in the standard-dose group.

### Subgroup Analyses

**Figure 3:**
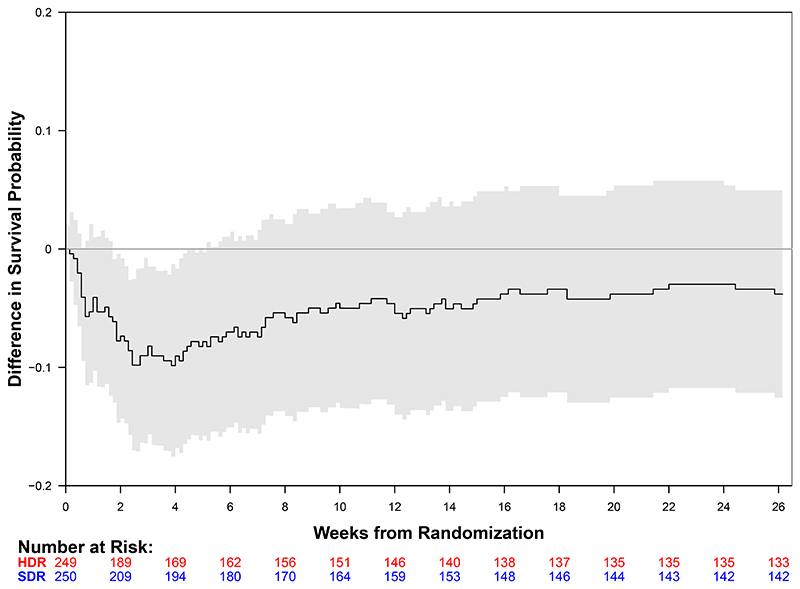

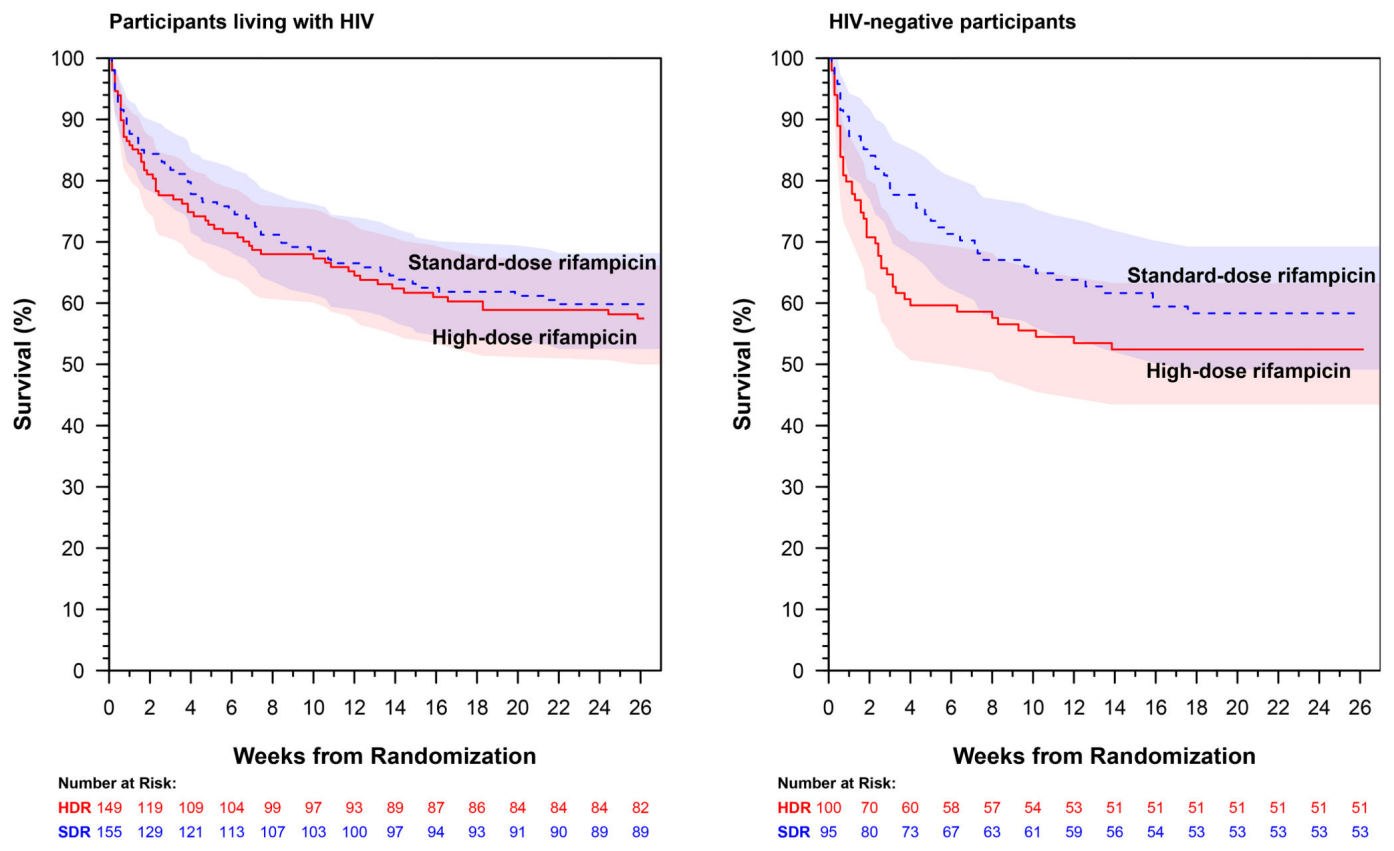

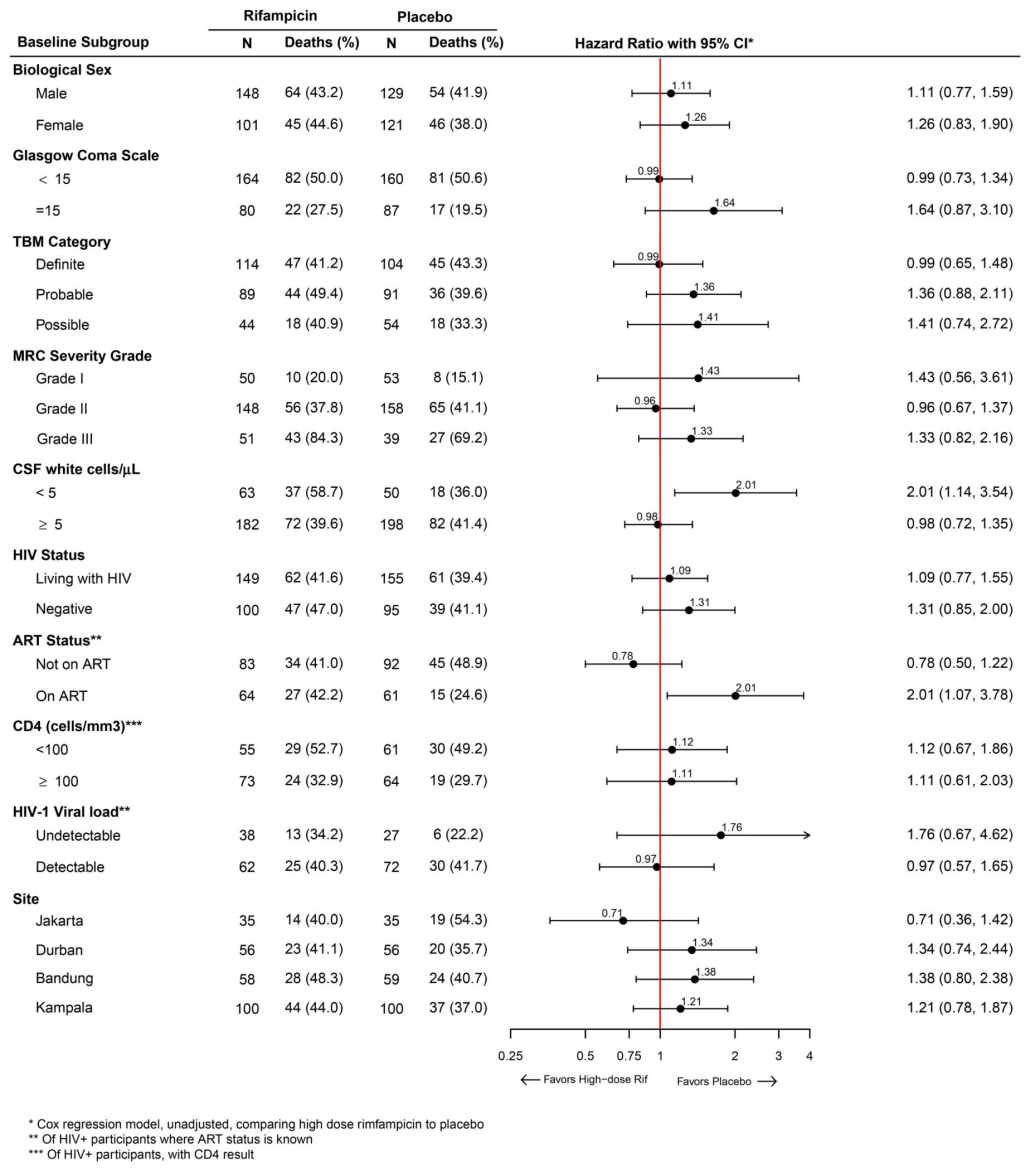
Forest Plot assessing treatment effect heterogeneity by baseline characteristics Interactions between the treatment group and prespecified baseline subgroups were assessed to determine whether the treatment effect (hazard ratio of high-dose rifampicin vs standard-dose placebo for death) were dependent on those apriori identified baseline characteristics. Subgroups were generally consistent with the overall outcome, none showing the benefit of high-dose rifampicin. Figures S1 in the Supplementary Appendix display Kaplan-Meier curves by subgroups. HIV viral suppression was selected as a post-hoc subgroup, based on the antiretroviral therapy finding.

None of the subgroup analyses showed evidence of beneficial effects of high-dose rifampin. (Figure 3). We observed higher risk for mortality in the high-dose group compared to the standard-dose group among those receiving antiretroviral therapy at the time of presentation (Hazard Ratio, 2.01; 95% CI, 1.07 to 3.78). Among participants with CSF white cells <5 cells/mm^3^, we observed higher risk for mortality in the high-dose group compared to the standard-dose group (Hazard Ratio, 2.01; 95% CI, 1.14 to 3.54) whereas the hazard ratio among those with ≥5 cells/mm^3^ was 0.98 (95% CI, 0.72 to 1.35).

### Secondary Outcomes

No evidence of benefit was observed for any secondary endpoints. First, in assessing functional and neurological disability, we observed no difference in the modified Rankin score (Odds Ratio, 0.80; 95% CI, 0.58 to 1.11) or Liverpool outcome score at 24-weeks (Table S4). Among participants who had baseline GCS <15, 48 of 169 (28%) had normalized their mental status by day 20 in the high-dose rifampin group, and 65 of 163 (39.9%) in the standard dose group achieved this. The median of Liverpool outcome score was 53 (interquartile rage: 1 to 72) among 204 participants in the high-dose rifampicin group and was higher in the standard-dose group with median 63 (interquartile range 41 to 73) among 202 participants at Week 2 (Table S5B). 12-month mortality did not differ between the high-dose (Kaplan-Meier estimate: 47.0%) and standard-dose groups (Kaplan-Meier estimate: 43.7%) (Hazard Ratio, 1.14; 95% CI 0.88 to 1.49, Figure S12).

There was no evidence for difference in safety between study arms. Study drug discontinuation for more than 5 days, mostly due to hepatotoxicity, was similar between the two groups (Table S6). Overall, 217 serious adverse events occurred among 185 participants. There was no evidence of difference in any of the clinical grade 3-5 adverse events between groups, except for aspiration pneumonia which occurred in 13 of 249 (5.2%) high-dose participants compared with 4 of 250 (1.6%) standard-dose participants. No difference was observed in grade 3-4 laboratory abnormalities between groups, except total bilirubin for which elevations were more frequent in high-dose participants (24 of 249 [9.6%] compared with standard-dose participants (9 of 250 [3.6%]) (Table S7). Drug-induced liver injury occurred in 20 of 249 (8.0%) participants in the high-dose group and 11 of 250 (4.4%) in the standard-dose group. The number of participants with an alanine transaminase measurement ≥5 times the upper limit of normal was similar in the two groups (Table S8). Blinded study drug was interrupted for >5 days due to drug-induced liver injury in 6 of 249 (2.4%) high-dose participants vs 4 of 250 (1.6%) standard-dose participants. No participants died of drug-induced liver injury.

The initial duration of hospitalization did not differ. Within the first six months, there were 101 re-hospitalizations, with stroke, other infections, and tuberculosis paradoxical reactions or immune reconstitution inflammatory syndrome as the main reasons (Table S9).

## Discussion

This randomized double-blind trial demonstrated no benefit of high-dose rifampicin as prescribed among adults with tuberculous meningitis with respect to 6-month survival or any prespecified secondary endpoints or subgroups. Although no excess drug toxicity occurred, higher early mortality and slower normalization of mental status were observed in the intervention arm. Two *a priori* subgroups had two-fold higher hazards of death with high-dose rifampicin as compared to standard-dose rifampicin therapy; those with CSF white cells <5 per mm^3^ and those with HIV receiving antiretroviral therapy at baseline.

The lack of benefit could have several possible explanations. First, lower exposure of adjunctive corticosteroids in the high-dose arm may have occurred due to greater induction of hepatic metabolism. One study has shown that rifampicin dosed at 40 mg per kilogram reduces exposure of midazolam, used as a probe drug for hepatic cytochrome p450 CYP3A which also metabolizes dexamethasone and prednisone, by 38% compared to standard-dose rifampicin.^20^ Measurement of corticosteroids in blood from study participants may shed further light on this. In support of this hypothesis, we observed high-dose rifampicin had a greater absolute 6-month mortality in HIV-negative participants (5.9% lower survival than standard-dose rifampicin) than in those with HIV (2.2% lower survival than standard-dose rifampicin). This is consistent with corticosteroids having a greater impact on tuberculous meningitis outcomes in patients without HIV (~7.2% absolute mortality reduction) than in those with HIV (~4.9% reduction).^3,21^ Thus, increased corticosteroid metabolism could have had a greater detrimental impact in participants without HIV.^3^ But, steroid metabolism alone would not explain the lack of survival benefit in persons with HIV.

Second, more rapid killing of mycobacteria with higher-dose rifampicin might theoretically lead to a stronger, and detrimental, immunologic reaction. This could potentially explain the lower survival with higher-dose rifampicin in those on antiretroviral therapy compared to those not receiving antiretroviral therapy (18.9% lower survival versus 8.4% higher survival), as those on antiretroviral therapy would have the ability to generate a more robust immunologic response. A dysregulated response in the central nervous system can be detrimental, as previously shown for earlier antiretroviral therapy after cryptococcal meningitis.^22,23^ Somewhat surprisingly, participants with CSF <5 white blood cells/mm^3^ at baseline, which might represent a dysfunctional or anergic immune response,^24^ had 22.7% higher mortality with high-dose rifampicin than standard-dose. The majority of participants with CSF white cells <5 cells/mm^3^ were living with HIV, but 30% were HIV-negative. Our ongoing immunological and multi-omics studies may shed light on this.

The rifampicin dosing choice is unlikely to explain the lack of effect. A previous phase III trial combining a more modest increased rifampicin dose of 15 mg per kilogram with levofloxacin showed no benefit in Vietnam.^2^ Subsequent, model-based pharmacokinetic-pharmacodynamic meta-analyses identified daily dosing of >30 mg per kilogram as optimal.^5^ High-dose rifampicin is being evaluated in four out of five ongoing tuberculous meningitis clinical trials, involving >1,800 adults and children.^25^ We believe our choice of testing 35 mg per kilogram dosing was reasonable and is close to the 40 mg per kilogram maximal tolerable dose.^26^ No excess toxicity associated with high-dose rifampicin occurred except for non-fatal drug-induced hepatotoxicity and transient benign hyperbilirubinemia likely caused by decreased bile efflux of conjugated bilirubin with high-dose rifampicin.^26^ Difference in hepatic transaminase elevation was not observed.

Our trial has several strengths. We examined a blinded, single intervention, enrolling adults with and without HIV across the spectrum of disease and in three countries on two continents. As such, our results are highly generalizable. Retention of participants, adherence to study drugs, and ascertainment of all trial endpoints were almost complete. The major limitation common to all tuberculous meningitis studies is the potential for misdiagnosis. Microbiologic confirmation of tuberculous meningitis is difficult, and any interventional therapy would not benefit patients who did not truly have tuberculosis. However, randomization should distribute any misdiagnoses equally between study arms. Overall, 80% had definite or probable tuberculous meningitis, by uniform case definition,^15^ and even those with definite tuberculous meningitis did not have a favorable trend for survival (Hazard Ratio 0.99). An additional limitation to interpreting our results is the limited follow-up CSF sampling over time. Future trials should prioritize longitudinal CSF sampling for biomarkers to help study possible underlying mechanisms for the differential effects related to CSF cell counts and antiretroviral therapy status we observed in this trial.

Our results raise the question of how the dismal outcomes can be improved. Rifampicin-free regimens consisting of potent antimicrobials with better brain penetration should be studied.^27^ Caution is warranted from any preclinical model, however, given that well-controlled murine model studies had shown clear benefit of high-dose rifampicin,^28^ underlining the complexity of this disease in humans and the importance of randomized clinical trials.

A second strategy might be host-directed strategies to target immunopathology.^1,29^ Adjunctive aspirin is being evaluated in several trials.^25^ Tumor necrosis factor antagonist infliximab has benefit for tuberculous meningitis patients with corticosteroid-refractory inflammatory events later in the course of antituberculous treatment,^30^ but as deaths and irreversible neurological damage mostly occur in the first few weeks, trials should evaluate infliximab at treatment initiation. Finally, interventions that shorten the complex and lengthy patient pathways^31^ or lead to earlier diagnosis will likely improve outcomes.^32,33^

Although high-dose rifampicin did not benefit adults with tuberculous meningitis, this trial demonstrates that high-quality multinational trials can be conducted successfully, despite the complexities of care. Our findings should stimulate more research for this neglected disease.

Disclosure forms provided by the authors are available with the full text of this article at NEJM.org.

## Figures and Tables

**Table 1:** Baseline Characteristics in the Intention-To-Treat Population Additional baseline HIV demographics are presented in Table S3 in Supplementary Appendix.

Characteristic	High-dose rifampicin (N=249)	Standard-dose rifampicin (N=250)
Median age (IQR), years	38 (28-46)	35 (28-45)
Female sex, no. (%)	101 (40.6)	121 (48.4)
Median weight (IQR), kg	54 (49-60)	55 (45-62)
MRC disease severity grade, no. (%)		
Grade 1	50 (20.1)	53 (21.2)
Grade 2	148 (59.4)	158 (63.2)
Grade 3	51 (20.5)	39 (15.6)
Glasgow Coma Scale <15, no. (%)	164 (67.2)	160 (64.8)
Living with HIV, no. (%)	149 (59.8)	155 (62.0)
On antiretroviral therapy, no. (%)	64 (43.0)	61 (39.4)
Median CD4 cell count (IQR), cells/mm^3^*	101 (38-176)	85 (43-204)
Tuberculosis treatment at enrolment, no. (%)	175 (70.3)	173 (69.2)
Median doses in last 7 days (IQR)	3 (1-4)	3 (1-4)
Diagnostic category		
Definite TBM	144 (45.8)	104 (41.6)
Probable TBM	89 (35.7)	91 (36.4)
Possible TBM	44 (17.7)	54 (21.6)
Not TBM ^†^	2 (0.8)	1 (0.4)
Median creatinine (IQR), mg/dL^‡^	0.70 (0.55-0.89)	0.68 (0.55-0.80)
Median sodium (IQR), mmol/L^§^	133.0 (128.0-137.0)	133.0 (128.0-138.0)
Median total bilirubin (IQR), mg/dL^¶^	0.5 (0.3-1.0)	0.5 (0.3-0.8)
Median alanine transaminase (IQR), U/L^‖^	25 (16-43)	24 (15-40)
Cerebrospinal fluid test results		
Median white blood cell count (IQR), cells/mm^3^**	50 (4-177)	59 (11-203)
White blood cells <5 cells/mm^3^, no. (%)**	63 (26.0)	50 (20.0)
Median protein (IQR), mg/dL^††^	135 (85-239)	140 (82-243)
Median glucose (IQR), mg/dL^‡‡^	41.0 (24.8-61.0)	41.4 (25.0-58.0)
Median CSF:plasma glucose ratio (IQR)^§§^	0.4 (0.2-0.5)	0.4 (0.2-0.5)

* Data were available for 226 participants with HIV; 113 in the high-dose and 113 in the standard-dose group.

† Participants with an alternate confirmed diagnosis identified >14 days after randomization.

‡ Data were available for 486 participants; 243 in the high-dose and 243 in the standard-dose group.

§ Data were available for 485 participants; 241 in the high-dose and 244 in the standard-dose group.

¶ Data were available for 473 participants; 238 in the high-dose and 235 in the standard-dose group.

|| Data were available for 480 participants; 238 in the high-dose and 242 in the standard-dose group.

** Data were available for 493 participants; 245 in the high-dose and 248 in the standard-dose group.

†† Data were available for 487 participants; 244 in the high-dose and 243 in the standard-dose group.

‡‡ Data were available for 493 participants; 244 in the high-dose and 249 in the standard-dose group.

§§ Data were available for 380 participants; 186 in the high-dose and 194 in the standard-dose group.

**Table 2:** Clinical adverse events and laboratory abnormalities

Adverse Event	High-dose rifampicin (N=249)	Standard-dose rifampicin (N=250)	P value
**Serious adverse events** ^1^			
Participants with any serious adverse event	84 (33.7)	101 (40.4)	0.12
Number of serious adverse event	102	115	
Number of serious adverse event probably /	7 (6.7)	5 (4.3)	
definitely related to treatment			
**Most frequently reported grade 3-5 adverse** **events** ^1^	*number of participants (percent)*		

Number of grade 3-5 adverse events	192	194	
Participants with ≥1 events	123 (49.4)	129 (51.6)	
Neurological			
Cerebrovascular accident	9 (3.6)	10 (4.0)	0.82
General seizures	5 (2.0)	6 (2.4)	0.77
Partial seizures	3 (1.2)	2 (0.8)	0.65
Space occupying lesion	3 (1.2)	2 (0.8)	0.65
Immune reconstitution inflammatory syndrome	5 (2.0)	4 (1.6)	0.73
Aspiration pneumonia	16 (6.4)	4 (1.6)	0.006
Sepsis			
Systemic inflammatory response syndrome without identified bacteremia	6 (2.4)	7 (2.8)	0.78
Sepsis with bacteremia	4 (1.6)	10 (4.0)	0.11
Shock with multiorgan failure	14 (5.6)	13 (5.2)	0.83
**Hepatic events - grade 3-4** ^1,2^	*number of participants (percent)*		
Alanine transferase (≥5x upper limit of normal)	13 (5.2)	15 (6.0)	0.71
Alkaline phosphatase (≥5x upper limit of normal)	0 (0)	0 (0)	-
Total bilirubin (≥2.6x upper limit of normal)	24 (9.6)	9 (3.6)	0.007
Drug-induced liver injury ^2^	20 (8.0)	11 (4.4)	0.09
Deaths related to drug-induced liver injury	0	0	-
Tuberculosis drug discontinuation for >5 days	6 (2.4)	4 (1.6)	0.52

1 See Table S7 in Supplementary Appendix for full serious adverse events listing, clinical and laboratory adverse events.

2 Protocol-defined criteria for drug-induced liver injury are ≥3 upper limits of normal with symptoms or ≥5x upper limit of normal without symptoms (Supplementary Methods).
